# When a Social Experimenter Overwrites Effects of Salient Objects in an Individual Go/No-Go Simon Task – An ERP Study

**DOI:** 10.3389/fpsyg.2018.00674

**Published:** 2018-05-17

**Authors:** René Michel, Jens Bölte, Roman Liepelt

**Affiliations:** ^1^Institute of Psychology, University of Münster, Münster, Germany; ^2^Institute of Psychology, German Sport University Cologne, Cologne, Germany

**Keywords:** Simon effect, EEG, joint action, action perception, referential coding, compatibility effect

## Abstract

When two persons share a Simon task, a joint Simon effect occurs. The task co-representation account assumes that the joint Simon effect is the product of a vicarious representation of the co-actor’s task. In contrast, recent studies show that even (non-human) event-producing objects could elicit a Simon effect in an individual go/no-go Simon task arguing in favor of the referential coding account. For the human-induced Simon effect, a modulation of the P300 component in Electroencephalography (EEG) is typically considered as a neural indicator of the joint Simon effect and task co-representation. Showing that the object-induced Simon effects also modulates the P300 would lead to a re-evaluation of the interpretation of the P300 in individual go/no-go and joint Simon task contexts. To do so, the present study conceptually replicated Experiment 1 from Dolk et al. (2013a) adding EEG recordings and an experimenter controlling the EEG computer to test whether a modulation of the P300 can also be elicited by adding a Japanese waving cat to the task context. Subjects performed an individual go/no-go Simon task with or without a cat placed next to them. Results show an overall Simon effect regardless of the cat’s presence and no modulatory influence of the cat on the P300 (Experiment 1), even when conceivably interfering context factors are diminished (Experiment 2). These findings may suggest that the presence of a spatially aligned experimenter in the laboratory may produce an overall Simon effect overwriting a possible modulation of the Japanese waving cat.

## Introduction

Coordinating human interaction is part of our daily life’s challenges. Even simple activities as carrying furniture together require precise coordination of one’s own action with our co-actors’ actions (van der Wel et al., 2016). Own produced actions and perceived actions are mentally represented in a functionally similar way (Prinz, 1997; Hommel et al., 2001), a perspective that follows from the logic of common coding between perception and action planning and control (Prinz, 1997). An often-used task to test the concomitant interplay between perception and action in a shared task context is a modified version of the *Simon task* (Simon and Rudell, 1967; Lu and Proctor, 1995).

In the standard Simon task, a participant is asked to respond on a non-spatial, dichotomous stimulus attribute (e.g., color: red/green) with two spatially arranged response buttons (e.g., horizontally: left/right) while ignoring a task-irrelevant spatial stimulus dimension (e.g., stimulus location: left/right). According to the dimensional overlap model (Kornblum et al., 1990), the stimulus location primes the spatially compatible response. This results in faster and more accurate responses for compatible trials (required response and stimulus on the same side) compared to incompatible trials (spatial location of response and stimulus differ) which will elicit a response conflict requiring additional time to be solved (De Jong et al., 1994; Nicoletti and Umiltà, 1994; Hommel et al., 2001). This compatibility effect is the so-called Simon effect (Simon and Rudell, 1967; Hedge and Marsh, 1975).

In a variant of the standard Simon task, the individual go/no-go Simon task, subjects are asked to respond only to one of the two stimulus attributes (e.g., respond to a green stimulus; do not respond to a red stimulus; Sebanz et al., 2003). Here, the Simon effect is typically absent (but see Stenzel and Liepelt, 2016b). It is argued that there is no stimulus-response compatibility because the actor’s response is not spatially coded (Hommel, 1996; Sebanz et al., 2003, 2006; Liepelt et al., 2011, 2013; Kiernan et al., 2012; Dolk et al., 2013a).

Sebanz et al. (2003) developed the joint Simon task to test the impact of another person’s action on one’s own task performance during joint action (Sebanz et al., 2003). Two participants performed a standard Simon task simultaneously, sitting side by side to each other. As in the individual go/no-go Simon task, each participant responded to only one of the two stimulus attributes (Sebanz et al., 2003, 2006). Although, when regarded separately, each participant performed an individual go/no-go Simon task (which normally does not elicit a Simon effect), the Simon effect re-appeared in this joint setup, therefore called joint Simon effect (Hommel et al., 2001; Sebanz et al., 2003, 2005, 2006; Tsai et al., 2006; Tsai and Brass, 2007; Kiernan et al., 2012; Welsh et al., 2013a).

Sebanz et al. (2003, 2006) explained the joint Simon effect by assuming an automatic representation of our co-actors’ actions and tasks. The task co-representation account implicates that merely seeing the stimuli relevant for a co-actor already activates the required action of our interaction partner based on the knowledge about his/her task rules stressing the social aspect of the effect (Tsai and Brass, 2007). As own and foreign actions are mentally represented in a functionally similar way (Sebanz et al., 2006), the concept of common coding (Prinz, 1997) is extended to entire tasks. The joint representation of both task shares (own plus other half of the Simon task), evokes a mental representation of an entire Simon task. Given this shared representation, a spatially driven stimulus-response compatibility effect emerges such as if, e.g., my left partner’s action is represented like my left response hand in the standard Simon task.

The task-co-representation account assumes that shared representations measured by the joint Simon effect reflects the basis for social interaction (Knoblich and Sebanz, 2006) as it was found to be mediated by social factors like group membership and cooperative or competitive relationship of the co-actors (Hommel et al., 2009; Ruys and Aarts, 2010; Iani et al., 2011).

However, studies with non-social set-ups, e.g., with robots or programmed wooden hands, are inconclusive with respect to the question if an interaction partner needs to be always socially encoded (Tsai and Brass, 2007; Müller et al., 2011; Stenzel et al., 2012, 2013; Stenzel and Liepelt, 2016b; Puffe et al., 2017). Furthermore, the size of joint and individual go/no-go Simon effects seems to depend on agency cues like human body form (Tsai and Brass, 2007), ostensive cues like turn taking characteristics of the response (Stenzel and Liepelt, 2016b), and the exact task conditions showing some dependence of stimulus modality (Lien et al., 2016; Puffe et al., 2017). Additional factors that influence the presence of a Simon effect in an individual go/no-go Simon task are related to the degree to which participants spatially code their responses (Dittrich et al., 2012, 2013). Enhanced spatial response coding may be achieved, for example by using different hand positions (Liepelt, 2014) or by responding with pointing actions (Porcu et al., 2016) as well as by decreasing the spatial proximity between two actors so that the other person’s action moves from extrapersonal space into the peripersonal space (Guagnano et al., 2010).

Due to the increased number of findings showing a Simon effect in individual go/no-go Simon task settings, a new account has been proposed – the referential coding account (Weeks et al., 1995; Vlianic et al., 2010; Dolk et al., 2011, 2013a,b). Its theoretical grounding is the theory of event coding – TEC (Hommel et al., 2001). According to TEC, a bundle of feature codes representing a combination of their attributes (e.g., spatial orientation, sound, color, form etc.) mentally represents actions. Based on early assumptions of ideomotor theory (Lotze, 1852; James, 1890), these feature codes resemble those perceptual events that typically follow the action in the outside world. The more attributes internal and external events share, the more likely they activate each other. High similarity between perceived events and events used for action control increases self-other integration (Prinz, 2005; Dolk and Prinz, 2016). The referential coding account explains the joint Simon effect by assuming a discrimination problem between externally perceived and internally activated events (Dolk et al., 2014): the higher the similarity between internal and external events is (i.e., the more features they share), the harder is the discrimination problem. To resolve it, an actor must focus on task features that best distinguishes own from other events in a given task context. Spatial orientation can serve as such a discriminating feature (Miller et al., 2011), but depending on task context other features such as color (Sellaro et al., 2015) or valence (Stenzel and Liepelt, 2016a) can be used as well to resolve the discrimination problem.

According to the referential coding account, individual go/no-go Simon effects occur when an event-producing object shares enough attributes with the participant’s action (e.g., a clicking sound representing an auditory effect of an action) and when two actors produce events in relative spatial proximity. Thereby, in principle the referential coding account is able to explain the presence of a joint Simon effect produced by a social co-actor and non-socially produced Simon effects produced by objects such as a Japanese waving cat or a metronome (Dolk et al., 2013a) parsimoniously by applying the same basic mechanism.

To investigate the neural mechanisms underlying the Simon effect, the EEG is an appropriate method providing a high temporal resolution (for an overview see Leuthold, 2011). The P300 is a positive component at parietal electrodes with a latency of 250 to 500 ms after stimulus onset. It serves commonly as a relative measure for stimulus evaluation (Kutas et al., 1977; Magliero et al., 1984; Kok, 2001), functioning as a mediator between perceptual analysis and response preparation (Verleger et al., 2005) as well as an indicator for action control (Fallgatter and Strik, 1999). Using visual stimuli in a standard Simon task, the stimulus-response compatibility has been shown to influence the amplitude and the latency of the P300 (Ragot and Renault, 1981; Magliero et al., 1984; Renault et al., 1988; Valle-Inclán, 1996; Zhou et al., 2004). Regarding individual go/no-go Simon tasks, no-go-trials in contrast to go-trials show larger amplitudes and longer latencies for the P300 which provides evidence for its involvement in response inhibition (Roberts et al., 1994; Falkenstein et al., 1995; Bokura et al., 2001; Tekok-Kilic et al., 2001).

Sebanz et al. (2006) investigated this no-go P300 effect in a joint Simon task contrasting a group condition (two participants in a joint Simon task) with an individual condition (one participant performing an individual go/no-go Simon task). Only in the group condition, a Simon effect was found. Further, a higher P300 amplitude on no-go-trials in the group as compared to the individual condition was interpreted as an indication of the joint Simon effect and task co-representation. To confirm the referential coding account’s postulation that human- and object-induced Simon effects have the same underlying mechanisms, a study with an object-induced Simon effect investigating the no-go P300 is needed. If the postulation is correct, the no-go P300 effect found by Sebanz et al. (2006) should also be observed at an object-induced Simon effect. For this investigation, the experimental setup used in Experiment 1 of Dolk et al. (2013a) qualifies best: they asked participants to perform an auditory individual go-no/go Simon task with or without sitting next to a Japanese waving cat. In contrast to the cat absent condition, a Simon effect occurred in the cat present condition.

Lien et al. (2016) already adopted this Japanese waving cat manipulation used in Experiment 1 by Dolk et al. (2013a) and added EEG recordings. In two experiments, subjects performed subsequently both a standard and a go/no-go Simon task with or without the cat placed next to them and with auditory (Experiment 1) or visual stimuli (Experiment 2). In contrast to Dolk et al. (2013a), Lien et al. (2016) used pitched tones instead of reversed Dutch words as auditory stimuli and red or green colored points presented within a picture of a hand pointing to the left, right or central direction as visual stimuli. Whereas a Simon effect was found for the standard Simon task independent from cat presence, for the go/no-go task a Simon effect was only observed in the cat present condition when using auditory stimuli but not when using visual stimuli. Regarding EEG, they found a modulation of the lateralized readiness potentials (LRPs) induced by the cat in the go/no-go task only for auditory stimuli. As they used LRPs as a neuronal indicator instead of the P300 used for human-induced Simon effects by Sebanz et al. (2006), the question whether object-induced Simon effects also elicit such a P300 effect still needs neuropsychological confirmation.

Thus, the present study has the objective to add this pending evidence by replicating Experiment 1 of Dolk et al. (2013a) adding EEG recordings to investigate the P300 because it was previously taken as an indicator for a joint Simon effect in humans (Sebanz et al., 2006). Thus, we tested if a Japanese waving cat elicits a joint Simon effect (cat present condition) and compared the participant’s performance to an individual go/no-go Simon task (cat absent condition). Additionally, we contrasted the P300 on no-go-trials in the cat present and cat absent condition. Deviating from Dolk et al. (2013a), visual instead of auditory stimuli were presented for a better comparability of the P300 with Sebanz et al. (2006).

Based on the referential coding account, we predict (1) a larger Simon effect in the cat present condition as compared to the cat absent condition and (2) a significantly increased (more positive) amplitude for the P300 component for the cat present condition compared to the cat absent condition in the collected EEG data. In contrast, based on the task-co-representation account, we predict (1) neither a behavioral Simon effect in cat present or cat absent conditions (2) nor a compatible/incompatible P300 difference corresponding to the Simon effect.

## Experiment 1

### Method

#### Participants

Twenty-four participants (12 female) at the age of 20 to 30, *M* = 23.08, *SD* = 2.22, took part in the Experiment^1^. Nineteen of them were psychology students. All participants were right-handed and had normal or corrected-to-normal vision. All participants gave their written informed consent to participate in the study, which was conducted in accordance with the ethical standards laid down in the World Medical Association Declaration of Helsinki (2013) and approved by the ethical committee of the University of Muenster. Participants with psychiatric diseases, heavy head injuries in the past or metallic cranial-implants were excluded with the help of a screening questionnaire. For participating students received course-credit for participation.

#### Material

**FIGURE 1:**
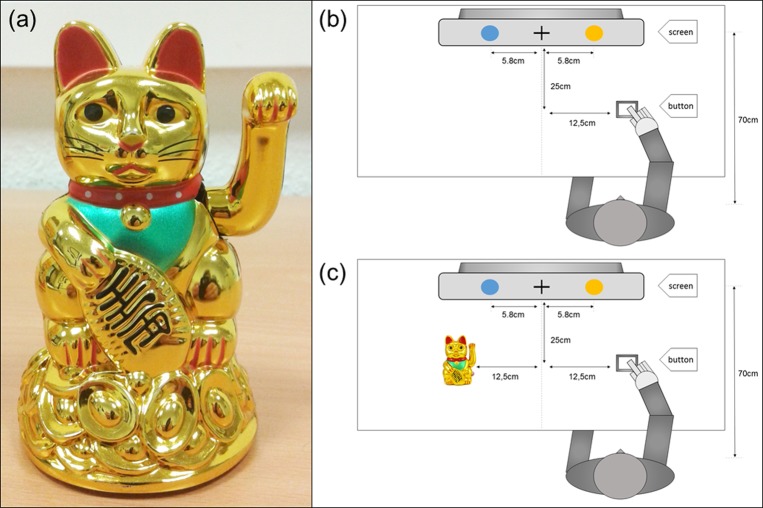
Arrangement with specifications. **(a)** Photo of the Japanese waving cat. **(b)** Setting cat absent condition. **(c)** Setting cat present condition.

The participant sat on a fixed chair in front of the right edge of the screen. A fixed button was placed in front of the participant. A Japanese waving cat (height: 12.5 cm, width: 9 cm, depth: 7 cm, see **Figure 1**) facing the subject was placed to the left of the participant in the cat present condition (for the entire task arrangement see **Figure 1**). The cat’s left arm waved at steady frequency of 0.4 Hz and movement angle of 50° in the vertical plane. While waving, the cat produced a steady clicking sound.

The participant was instructed to place the right hand flatly on the table while putting the index finger on the button. The left hand was placed on the left upper leg during the whole experiment. The laboratory was slightly dimmed, the examiner controlling the EEG measurement was positioned out of the participant’s field of view two meters away on the left side.

#### Procedure

Participant’s task was to push the button as quickly and accurately as possible only when a blue dot (diameter = 2.2 cm) was presented. A yellow dot (diameter = 2.2 cm) was used as stimulus in the no-go-trials.

After eight warm-up trials, the genuine experiment with eight blocks containing 64 trials each was initiated. Each block consisted of 32 go- and 32 no-go-trials, with half of the trials being response-compatible (stimulus on the right side of the screen) or response-incompatible (stimulus on the left side of the screen), respectively. Within each block the trial sequence was randomized. Subsequent to each block, there was a short break of 1.5 min. The cat was presented randomly but counterbalanced over all participants either in the first or second half of the experiment. Preceding both cat present and cat absent condition, there was an instruction which only differed in introducing the Japanese waving cat.

Each go-trial started with the sole presentation of a fixation cross (200 ms, 0.6 cm × 0.6 cm). Then, along with the fixation cross, a blue dot (to the left or right of the fixation cross, distance = 5.8 cm) was presented for 500 ms. If the response button was pressed within the 500 ms, fixation cross and the blue dot disappeared immediately. Each no-go-trial started with the sole presentation of a fixation cross for 200 ms followed by the combined presentation of the fixation cross and a yellow dot to the left or right of it (distance = 5.8 cm). The yellow dot’s initial presentation duration was 350 ms and then was adjusted to the participant’s reaction time (RT) by setting of the preceding no-go stimulus presentation duration off against the participant’s last RT.

Time out was set to 1000 ms and the participant received the feedback “too slow.” False positive answers led to the feedback “mistake.” The whole procedure took about 40 min. Finally, participants completed a questionnaire targeting in how far the Japanese waving cat attracted the participants’ attention or was perceived as an object (instead of manlike).

#### EEG Measurement

EEG was recorded with *ASA*© (Advanced Source Analysis, ANT Neuro, Enschede, Netherlands) with a 32-electrode configuration of a 64 ANT-Waveguard cap (10–20 system). Resistance was kept below 5 kΩ. The signal was amplified (ExG 20x, fixed = 5 mV/V) and recorded continuously during the whole experiment with an average reference and a lowpass-Butterworth-filter (half-power cut off = 0.27 × sampling frequency) and a sampling frequency of 256 Hz. Vertical EOG was measured by placing a bipolar electrode beneath and above the left eye. Horizontal EOG was measured by placing a bipolar electrode at the outer canthus of each eye. AFz was used as ground electrode.

#### EEG Preprocessing

The continuous data was filtered in *ASA*© (version 4.8.1) with a half-power Butterworth-bandpass filter (0.1–20 Hz, 24 db/oct) based on the FFT-method. Noisy channels were interpolated. For artifact correction, a principal component analysis (PCA; Ille et al., 2002) was implemented based on manually marked artifacts. In *eeglab* (version 12.0.2.06b, *MATLAB* R2012b) the signal was down-sampled to 128 Hz sampling frequency and re-referenced to the mastoid electrodes (M1 and M2). The signal was epoched (200 ms before, 500 ms after stimulus onset) along with a baseline correction (200 ms before stimulus onset). Epochs with artifacts (threshold =±75 μV) were excluded. In *erplab* (version 4.0.2.3) only errorless and artifact-free epochs were averaged to event-related potentials (ERPs) separately for each condition.

### Results

Two participants had to be excluded from all further analyses (one because the mean RT was twice as high as for the rest of the participants, the other one due to EEG recording problems) leading to a sample size of 22 participants.

#### Behavioral Measurement

*R* (version 3.3.2) was used for statistical analysis. Analysis of error rates showed a mean error rate below 1% (for a detailed overview of RTs and error rates see **Table 1**); all error related trials were excluded from further analysis. For the following analysis, trimmed means (10% trim) of RT of correct go-trials were taken as dependent variable.

**Table 1:** Mean reaction time (RT) in milliseconds (trimmed 10%) and errors rates (ER) in percentages per cat presence, compatibility and experiment.

			Cat presence		
Experiment	dv	Compatibility	Cat absent	Cat present	*M*
1	RT	Compatible	298 (16)	296 (14)	297 (18)
		Incompatible	303 (12)	301 (14)	302 (16)
		*M*	301 (18)	299 (18)	
	ER	Compatible	0.00	0.00	0.00
		Incompatible	0.00	0.00	0.00
		*M*	0.00	0.00	
2	RT	Compatible	575 (28)	567 (30)	571 (36)
		Incompatible	584 (31)	571 (29)	577 (37)
		*M*	579 (36)	569 (35)	
	ER	Compatible	0.01	0.01	0.01
		Incompatible	0.01	0.01	0.01
		*M*	0.01	0.01	

*Error rates were calculated as percentage of all 512 trials. Standard deviations for RTs are shown in parentheses*.

**FIGURE 2:**
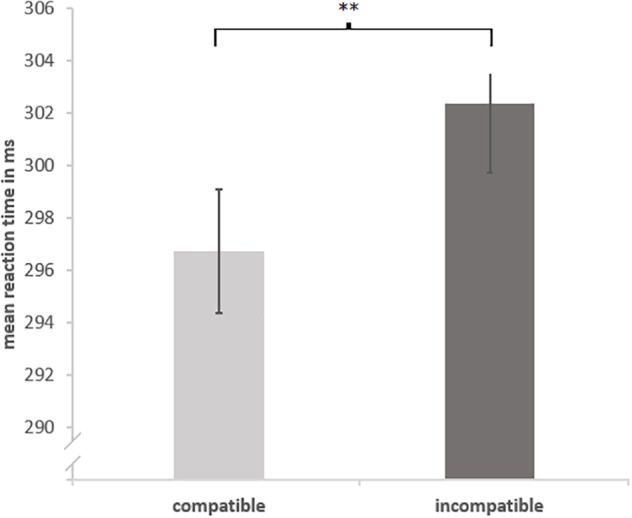
Main effect compatibility. Mean reaction times split by compatibility. Error bars show standard error corrected for within-subject designs (Morrey, 2008). “^∗∗^” shows *p* < 0.01.

For an analysis of variance for repeated measures (ANOVA) mean RT (10% trim) were calculated for each combination of the variables *compatibility* (compatible vs. incompatible) and *cat presence* (cat present vs. cat absent). An ANOVA^2^ including the within-subject factors *compatibility* and *cat presence* showed a significant main effect for *compatibility*, *F*(1,21) = 7.59, *p* = 0.01, ${\text{η}}_{\text{g}}^{2}$ = 0.01 (see **Figure 2**) with a facilitation for compatible trials, *M* = 297 ms, compared to incompatible trials, *M* = 302 ms (compatibility effect = 6 ms). The interaction *compatibility* ×*cat presence* was not significant, *F*(1,21) < 1.

#### EEG Analysis

For seven participants 1–4 channels were interpolated. Based on artifact detection for the preprocessed data, on average 0.8% of the trials per participant had to be excluded, *SD* = 1.79, maximum = 6.8%. Remaining trials were averaged to ERPs across the factors *compatibility*, *cat presence* and *go/no-go*.

**FIGURE 3:**
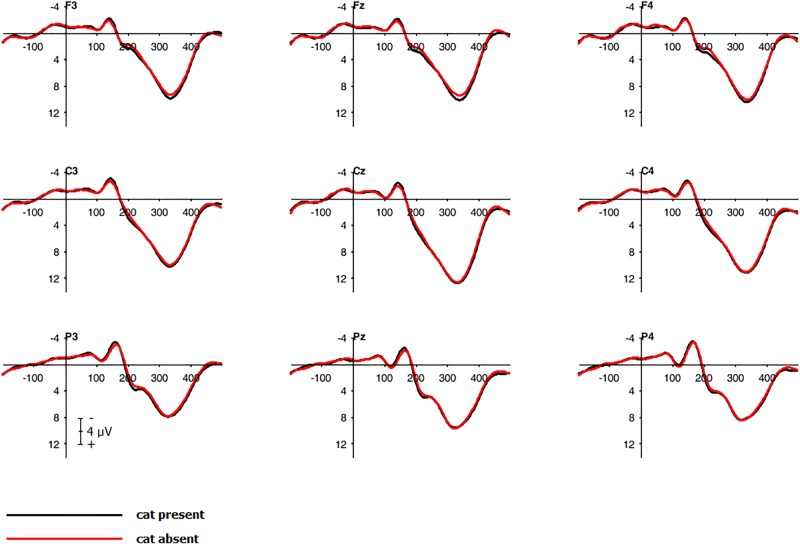
Grand average waveforms. Electrophysiological response to no-go-trials for frontal, central and parietal electrodes with and without the Japanese waving cat.

To investigate the main effect of *cat presence* on the P300 component for no-go-trials, a repeated measure, two-tailed cluster-based permutation test was calculated for a time window from 300 to 500 ms after stimulus onset. There were 2500 random permutations for each participant (Bullmore et al., 1999; Groppe et al., 2011). This resulted in 1530 tests (over 30 electrodes and 51 time points). To access an overall alpha-level of 0.05, a test wise alpha-level of 0.00033 was applied. Electrodes were considered as spatial neighbors within a radius of approximately 5.44 cm leading to clusters with a mean of 2.7 neighboring electrodes, *SD* = 1.2. The main effect *cat presence* for no-go-trials was not significant, *p*-values ≥ 0.56 (see **Figure 3** for corresponding waveforms). The same tests were calculated for the main effect *cat presence* for both go- and no-go-trials within an interval from 0 to 500 ms to cover the whole epoch leading to 3840 comparisons with a test wise alpha-level of 0.00013, but no cluster reached significance, *p*-values ≥ 0.21.

**FIGURE 4:**
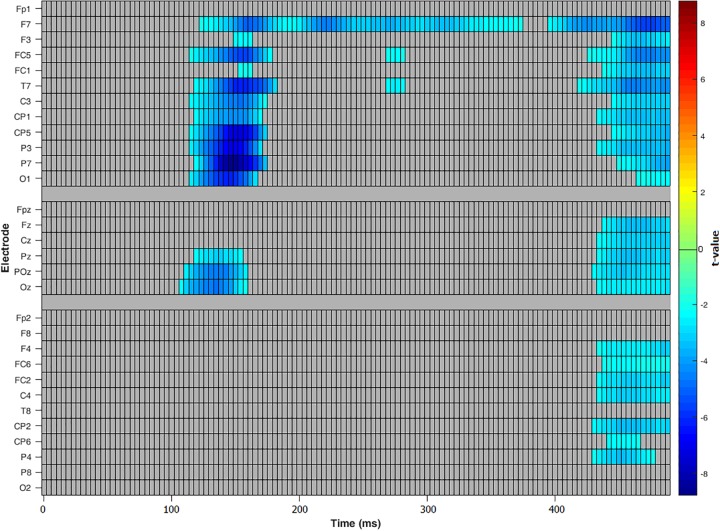
Cluster based permutation tests over all electrodes for main effect compatibility. Color key shows significant *t*-values for each electrode and time point with negative scores representing a higher amplitude for incompatible trials than for compatible trials.

**FIGURE 5:**
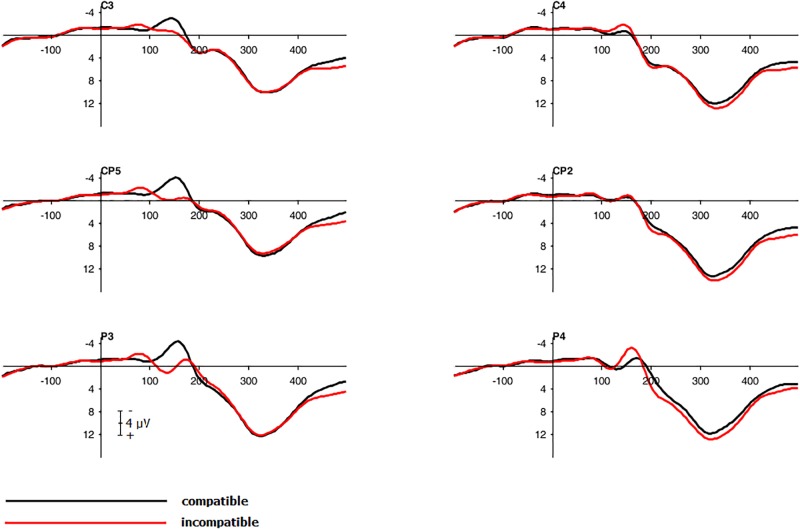
Grand average waveforms. Electrophysiological response to compatible and incompatible trials for central, centro-parietal and parietal electrodes.

To investigate a main effect of *compatibility*, a repeated measure, two-tailed cluster-based permutation test was calculated as described above with the following changes: To detect compatibility effects at all stages of the reaction process, an interval from 0 to 500 ms after stimulus onset was used leading to 3840 comparisons (over 30 electrodes and 128 time points). To access an overall alpha-level of 0.05, a test wise alpha-level of 0.00013 was applied. The main effect of *compatibility* was significant with higher amplitudes for incompatible trials. The effect was present in the entire left hemisphere within a time interval of 100 to 150 ms. The peak was located in the parietal and centro-parietal area with the smallest significant *t*-value *t*(21) = -2.09 and significant corrected *p*-values of 0.0016 (see **Figures 4**, **5**). The antagonistic effect in the right hemisphere with larger amplitudes for compatible trials than for incompatible trials within the same time window did not reach significance.

### Discussion

Experiment 1 of the present study aimed to replicate the object-induced joint Simon effect found by Dolk et al. (2013a) and the P300 effect induced by a human co-actor (Sebanz et al., 2006) using an individual go/no-go Simon task with visual stimuli.

Behavioral data showed a (1) main effect of compatibility with faster RTs for compatible trials than for incompatible trials but (2) no significant interaction between *cat presence* and *compatibility.* Regarding the ERPs, there was also a (3) main effect of *compatibility* located in the (centro-) parietal left hemisphere within a time interval of 100 to 150 ms with larger amplitudes for incompatible trials than for compatible trials. Regarding no-go-trials, there was (4) no significant modulation effect of *cat presence* on the P300 component.

The (1) main effect of *compatibility* with faster RTs for compatible trials than for incompatible trials prompts the presence of a Simon effect (Simon and Rudell, 1967) in the *cat present* as well as in the *cat absent* condition. The EEG data provide a neuronal correlate for this omnipresent Simon effect in form of the (3) early main effect of *compatibility* located in the (centro-) parietal left hemisphere. A similar early activation pattern for a compatibility effect was found by Valle-Inclán (1996) as well as Wascher and Wauschkuhn (1996) using a standard Simon task. In the present study, the Simon effect was not modulated by the Japanese waving cat, which was indicated by the non-significant interaction of *cat presence* and *compatibility* (2). The compatibility effect was not moderated by any effect of *order of presentation*. In accordance with our behavioral findings, there was (4) no significant modulatory effect of *cat presence* on the P300 for no-go-trials in the EEG data. In summary, the Japanese waving cat failed to modulate action inhibition despite the presence of an overall compatibility effect in an individual go/no-go Simon task with visual stimuli, which was contrary to our prediction.

Why did we observe a Simon effect, even in the cat absent condition without the Japanese cat? Neither the referential coding account (Dolk et al., 2013a), nor the task-co-representation account (Sebanz et al., 2006) can easily explain this pattern of Simon effects. While referential coding can explain the finding of a Simon effect in the *cat presence* condition, but not in the absence condition, task-co-representation fails to explain the finding of a Simon effect in both conditions because of a missing social co-actor.

One might speculate that the time-taking for preparation of the EEG measurement executed by the examiner and the examiner’s presence throughout the whole experiment could have evoked some kind of examiner effect. The examiner was located two meters left of the participant to control the EEG recording on a separate computer executing some mouse clicks or taking notes, which may have served as visual or auditory events that attracted the actor’s attention. If so, according to referential coding, one would need to assume that the presence of the experimenter’s actions on the participant’s left side must have forced the participant to spatially code one’s own action as right throughout the entire experiment, which may have been a stronger effect as of the presence of the Japanese cat itself.

Another explanation may arise from the no-go-stimuli’s presentation time: Sebanz et al. (2006) and Dolk et al. (2013a) worked with fixed presentation times matched to the maximum presentation time of go stimuli. Keep in mind that the go stimuli presentation duration is often shorter than the maximum presentation duration because the stimulus disappears as soon as the participant reacts. In the present study, the presentation time of no-go-stimuli was matched to the participant’s RT in go-trials to achieve a better comparability of go and no-go-trials. This matching might have changed the task structure for participants.

The cross modality of the visual stimuli and the primarily auditory events produced by the Japanese waving cat might also have influenced task performance. Dolk et al. (2011) used auditory stimuli not causing any cross modality. In addition, participants had to discriminate between auditory go and no-go-stimuli shifting attention to the auditory system. Discriminating auditory events (between the clicking sound produced by the cat and one’s own button press) was mandatory for task achievement. In the present study, we used visual stimuli, which might have taken attentional resources away from the visual events produced by the Japanese cat undermining its modulatory effect. This may explain why the cat did not further modulate the Simon effect.

Despite the shortcomings of the above explanation, we changed the paradigm to investigate the influence of the aforementioned problems. We tried to reduce as much as possible (1) the spatial coding of the examiner in our EEG task context, (2) used a fixed stimulus duration for the no-go-trials and (3) shifted from visual stimuli to auditory stimuli.

## Experiment 2

Experiment 2 was a replication of Experiment 1 introducing some minor changes aimed to more closely adopt the experimental setup to the study of Dolk et al. (2013a). As Dolk et al. (2013a) found a significant impact of a Japanese waving cat on the Simon effect, which we did not in Experiment 1 using visual stimuli, we performed the following changes to our task setup. We reduced the examiner’s influence to a minimum by screening off the examiner by a curtain from the participant’s room. Further, in line with the study of Dolk et al. (2013a), the no-go-stimulus presentation duration was no longer matched to the participant’s RT now using auditory stimuli.

### Method

#### Participants

Twenty-four participants (19 female) at the age of 18–52 years, *M* = 22.92, *SD* = 7.19, took part in the Experiment. Sixteen of them were psychology students. Screening procedure and participants’ payment was as equal to Experiment 1.

#### Material

**FIGURE 6:**
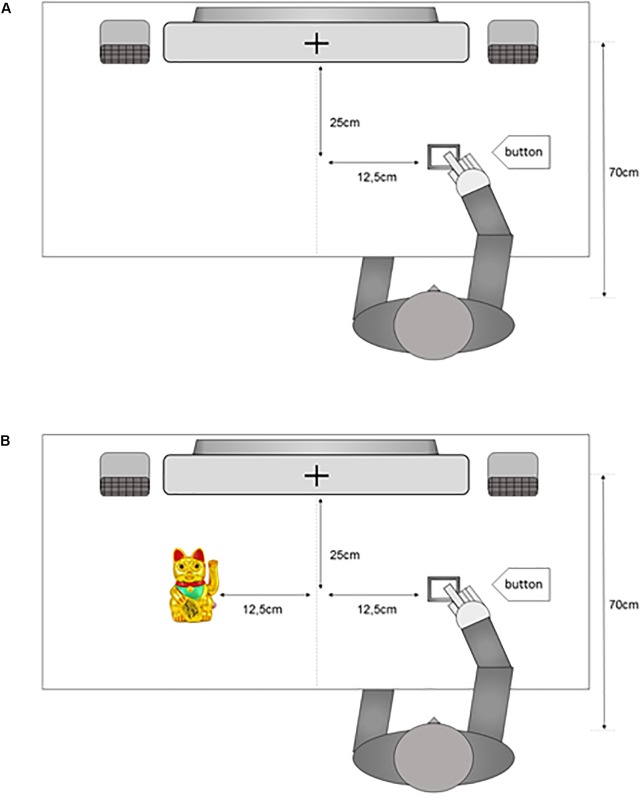
Arrangement with specifications. **(A)** Setting cat absent condition. **(B)** Setting cat present condition.

The experimental arrangement was identical to Experiment 1 expect the following changes: Two near field studio monitors *M-Audio AV32* were placed left and right to the screen (see **Figure 6**). Additionally, the examiner sat on the participant’s left side behind a noise-absorbing curtain completely screening off the examiner from the participant.

#### Procedure

The experimental procedure was identical to Experiment 1 expect the following changes: the participant had to press the button as quickly and accurately when the target sound was presented. No reaction was required for the no-go stimulus. Time-reversed versions of the spoken Dutch words *paars* or *groen* were used as target or distractor, respectively, counterbalanced over participants.

Each trial started with the presentation of a fixation tone (80 ms) and a fixation cross in the center of the screen (200 ms, 0.6 cm × 0.6 cm). Then, along with the fixation cross, the target or distractor sound was presented via the left or the right speaker for 300 ms. If the response button was pressed within the 300 ms, fixation cross and target sound disappeared immediately.

#### EEG Measurement and Preprocessing

Both measurement and preprocessing was implemented in the same way as already outlined in Experiment 1.

### Results

Three participants had to be excluded from all following analyses. One of them due to a high mean error rate of 11% (compared to 1% of the rest of the sample), while the other two had to be excluded due to recording problems. This led to a sample size of 21 participants.

#### Behavioral Measurement

*R* (version 3.3.2) was used for statistical analysis. Analysis of error rates showed a mean error rate of 1% (see **Table 1**); all error related trials were excluded from further analyses. Mean trimmed RT (10% trim) of RTs of correct go-trials served as dependent variable in the following analyses (see **Table 1**).

**FIGURE 7:**
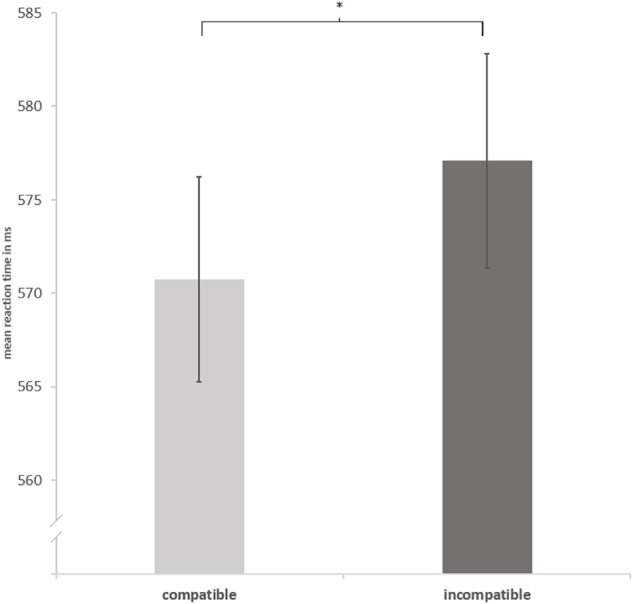
Main effect compatibility. Mean reaction times separated into compatibility. Error bars show standard error corrected for within-subject designs (Morrey, 2008). “^∗^” shows *p* < 0.05.

As in Experiment 1, mean RTs were submitted to an ANOVA^3^ including the within-subject factors *compatibility* (compatible vs. incompatible) and *cat presence* (cat present vs. cat absent). This analysis showed a significant main effect of *compatibility, F*(1,20) = 7.12, *p* = 0.015, ${\text{η}}_{\text{g}}^{2}$= < 0.01 (see **Figure 7**) with faster RTs for compatible trials, *M* = 571 ms as compared to incompatible trials, *M* = 577 ms (compatibility effect = 6 ms). The interaction *compatibility* ×*cat presence* was not significant, *F*(1,20) = 2.79, *p* = 0.11.

#### EEG Analysis

Data of one participant required interpolation of one channel. Based on artifact detection for the preprocessed data, on average 1.1% of the trials per participant had to be excluded, *SD* = 1.79, maximum = 7.5%. Remaining trials were averaged to ERPs across the factors *compatibility*, *cat presence*, and *go/no-go*.

**FIGURE 8:**
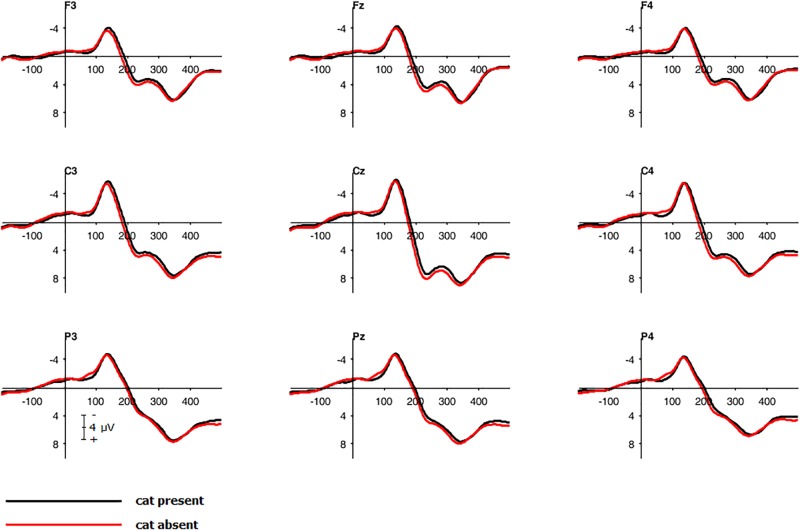
Grand average waveforms. Electrophysiological response to no-go-trials for frontal, central and parietal electrodes with and without the Japanese waving cat.

To investigate the main effect of *cat presence* on the P300 for no-go-trials, similar to Experiment 1, a repeated measure, two-tailed cluster-based permutation test was calculated for a time window from 300 to 500 ms leading to 1530 tests (over 30 electrodes and 51 time points) with an overall alpha-level of 0.05 by establishing a test wise alpha-level of 0.00033. Definition of electrode neighbors and clusters was parallel to Experiment 1. The main effect *cat presence* was not significant (*p*-values ≥ 0.42; see **Figure 8** for corresponding waveforms). The same tests as before were calculated separately for go and no-go-trials within an interval from 0 to 500 ms to cover the whole epoch leading to 3840 comparisons with a test wise alpha-level of 0.00013. These tests did not reach statistical significance either, no significant *t*-score, *p*-values ≥ 0.08. Thus, there was no effect of *cat presence* for go- or no-go-trials.

**FIGURE 9:**
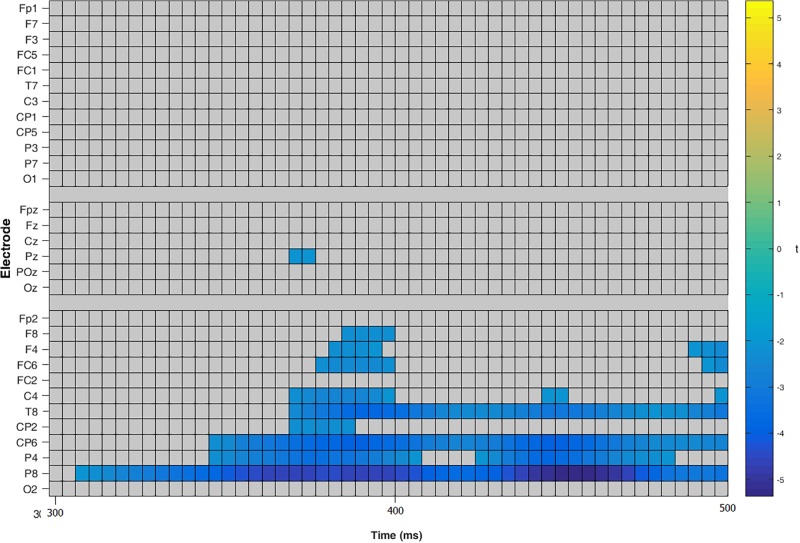
Cluster based permutation tests over all electrodes for main effect compatibility. Color key shows significant *t*-values for each electrode and time point with negative scores standing for a higher amplitude for incompatible trials.

**FIGURE 10:**
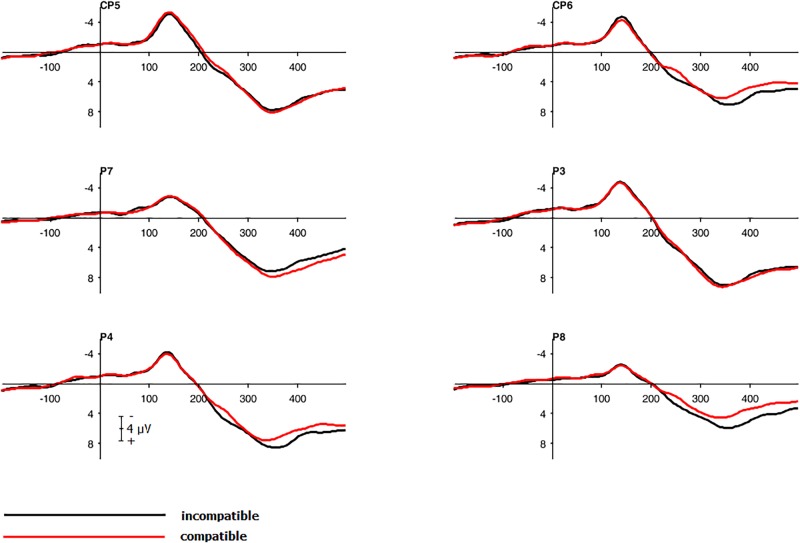
Grand average waveforms. Electrophysiological response to compatible and incompatible trials for centro-parietal and parietal electrodes.

To analyze the main effect of *compatibility*, a repeated measure, two-tailed cluster-based permutation test was calculated: An interval from 300 to 500 ms after stimulus onset was used leading to 1530 comparisons (over 30 electrodes and 51 time points) with an overall alpha-level of 0.05 by establishing a test wise alpha-level of 0.00003. The main effect *compatibility* was significant with higher amplitudes for incompatible trials than for compatible trials. The difference was evident in the right hemisphere in the parietal and centro-parietal area within an interval of 100 to 150 ms, smallest significant *t*-value *t*(20) = -2.09, significant *p*-values < 0.05 (see **Figures 9**, **10**).

### Discussion

Experiment 2 was conducted for conceptual replication of Experiment 1 using an optimized task design. It served to investigate whether a joint Simon effect (Sebanz et al., 2006) is evoked by a Japanese waving cat. Furthermore, the underlying neurophysiological processes were registered using EEG.

Similar to Experiment 1, there was an (1) overall Simon effect with faster response times for compatible trials than for incompatible trials regardless of the presence or absence of the Japanese waving cat. The predicted (2) interaction effect between *cat presence* and *compatibility* was not found. Additionally, a (3) *compatibility* effect was found in EEG. In contrast to Experiment 1, this effect was located in the (centro-) parietal right hemisphere within a later time window of 300–500 ms after stimulus onset and not in the left hemisphere as in Experiment 1. Furthermore, there was no (4) significant P300 effect regarding no-go-trials.

The finding of an (1) overall Simon effect suggests that the adapted paradigm, namely fixing the no-go-trials’ presentation time, screening off the examiner by a curtain as well as changing the stimulus modality did not affect the Simon effect. This suggests that despite the presence of the curtain the knowledge about the presence of the experimenter was enough to produce referential coding. This would be in line with studies showing evidence for a joint Simon effect when the two actors are seated in different rooms (Tsai et al., 2008; Ruys and Aarts, 2010) and the spatial arrangement of the two rooms allows a spatial coding of responses (Sellaro et al., 2013). Alternatively, or in addition, other factors of our setup may also contribute to a spatial coding of one’s own action. The (2) absence of the interaction of *cat presence* and *compatibility* shows that cat presence had no further modulatory influence on task performance. The (3) compatibility effect observed in a different location and later time window compared to Experiment 1 can be understood as a neurophysiological correlate of the Simon effect. The different location and time window is best explained by the change in stimulus modality from visual to auditory stimuli. The (4) missing modulation of the P300 effect for no-go-trials in the EEG-data, however, fits to the overall Simon effect and the missing interaction of cat presence and compatibility and provides ERP evidence that no modulation due to the Japanese waving cat took place.

## General Discussion

In this study, we performed two experiments replicating previous research on individual go/no-go Simon effects (Dolk et al., 2013a) to investigate the ERP effects underlying object induced Simon effects. Sebanz et al. (2006) reported an enhanced P300-effect in no-go-trials when two participants shared a Simon task as when the same go/no-go task was performed alone. We aimed to find a similar P300-enhancement when a go/no-go Simon task was performed next to a Japanese waving cat as when the task was performed alone.

In Experiment 1, we observed a Simon effect regardless of the presence or absence of the Japanese waving cat. Along with this, an early compatibility effect located in the (centro-) parietal left hemisphere was registered in the EEG data. A further modulation of the P300 component elicited by the Japanese waving cat was not observed.

As the influence of the cat might have been obscured by situational factors, in Experiment 2 the examiner was screened off with the help of a curtain, the modality was changed from visual to auditory stimuli and the presentation time of no-go-stimuli was no longer matched to the participant’s go-RT. These changes led to a Simon effect independent from the presence or absence of the Japanese waving cat. As in Experiment 1, there was a comparable compatibility effect present in the EEG. It differed from the EEG in Experiment 1 by a later onset of the compatibility effect and a different scalp location. We attribute this difference to the change in stimulus modality in Experiment 1 (visual) and Experiment 2 (auditory). Similar to Experiment 1, there was no significant P300 effect modulated by the presence of a Japanese waving cat.

The explanation of these findings leads to two main questions: which factors elicited a Simon effect independent from the presence or absence of the Japanese waving cat in the current two experimental settings? Why did we not find a clear modulation of the P300 by the Japanese waving cat?

The lack of a modulation of the P300 by the object may be understood when taking the voltage differences in the P300 between object absent and present condition as an indicator for an object-induced Simon effect (Sebanz et al., 2006). This procedure is based on the prerequisite that a Simon effect is absent in the object absent condition and present in the object present condition. As ruled out before, this requirement was not met in the present study. We clearly found a significant Simon effect in both object present and object absent conditions. The missing modulation of the P300 is therefore in line with the finding of an overall Simon effect observed for RTs and indicates that the cat had no modulating influence on the Simon effect in the present study.

This finding partly matches to recent results from Lien et al. (2016). The Lien study only found a significant modulation of the LRP by a Japanese waving cat when auditory stimuli were presented, but not for visual stimuli. Nevertheless, this only matches our findings of Experiment 1 where visual stimuli were used. The question remains, why we did not find a modulation of the Simon effect by cat presence when auditory stimuli (Experiment 2) were used.

On a behavioral basis, the interpretation that the visual stimulus modality may have diminished the modulating influence of the Japanese waving cat is supported by recent findings of Lien et al. (2016) and Puffe et al. (2017) who also replicated Experiment 1 by Dolk et al. (2013a) with a hidden or visible cat and with visual and auditory stimuli. Both studies found no modulation of cat presence with visual stimuli but a significant modulation when auditory stimuli were used. Our experiments fit into this pattern for Experiment 1 (visual stimuli), but not for Experiment 2 (auditory stimuli). Therefore, it remains unclear which factors elicited a Simon effect independent from the presence or absence of the Japanese waving cat in our study?

By adopting the task setup to the study of Dolk et al. (2013a), stimulus modality and the presentation times of the no-go stimuli could be ruled out as possible explanations for the missing effect of cat presence in Experiment 1. However, due to the EEG setup we used the impact of the experimenter could not fully be prevented. According to Tsai and Brass (2007), one factor modulating a joint Simon effect is the presence of a responding social co-actor. The only additionally present person in our study was the experimenter.

While the experimenter might have caused the Simon effect in Experiment 1, sitting two meters away on the left side, we tried to reduce his influence to a minimum by screening him off with a curtain in Experiment 2. As we also found a Simon effect in Experiment 2, it seems that even when placed behind a curtain in extra-personal space, the experimenter might have an impact on the spatial response coding for the participants, which would be in line with previous studies (Tsai et al., 2008; Ruys and Aarts, 2010; Sellaro et al., 2013). Our finding of a Simon effect when the experimenter was located in extra-personal space (in Experiment 2) is contrary to those of Guagnano et al. (2010) who did not show a joint Simon effect when two co-actors were located outside of peri-personal space (i.e., in extra-personal space) but support studies of Welsh et al. (2013a,b) showing a joint Simon effect when two co-actors were located in extra-personal space.

In addition to these previous studies, our findings seem to show that the exact task of the person placed behind the curtain is not relevant to induce a Simon effect in an individual go/no-go Simon task setting. One should be aware that a person sitting directly next to the participant simply observing the task does not elicit a joint Simon effect (Sebanz et al., 2003). Furthermore, our findings are in line with studies showing that it is not only relevant what we actually perceive of other persons actions, but what we imagine what other persons might be doing even when we cannot see them (Sellaro et al., 2013). The EEG experimenter represents a socially acting person being in the same room as the experimenter making it likely to catch attention. However, this person is clearly not involved in taking over the other half of the Simon task as it is the case in typical joint Simon tasks. Therefore, we do not think that action or task co-representation can account for the finding of the overall Simon effect we observed.

However, a weaker form of social attention might be involved in the effect we observed. In line with this assumption, we would therefore argue that perceiving an event-producing experimenter (Experiment 1) or imagining an event-producing experimenter (Experiment 2) is enough to induce referential coding and the Simon effect (Sellaro et al., 2013; Dittrich et al., 2017; Klempova and Liepelt, 2017).

This would also be in line with the findings of Puffe et al. (2017) who not only investigated if a Japanese waving cat next to the subject can elicit a Simon effect, but who also implemented a condition in which the cat was hidden behind a speaker so that it cannot be seen but only heard. This condition is somehow comparable to our approach to screen off the experimenter behind a curtain so he could not be seen but only heard (Experiment 2). As the experimenter had to produce some events while controlling the EEG recordings, he might have functioned in a similar way as the hidden but sound-producing Japanese waving cat in Puffe et al. (2017). As the hidden cat elicited a Simon effect when auditory stimuli were used, this might also be a suitable explanation for the overall Simon effect in our Experiment 2.

The assumption of attention induced effects fits to our finding that the omnipresent Simon effect in both experiments amounted to six or seven milliseconds, respectively. This effect size is not comparable to compatibility effects elicited by a standard Simon paradigm (approximately up to 26 ms, Simon and Rudell, 1967) but it is comparable to joint Simon effects (ranging between 7 and 15 ms, Kiernan et al., 2012; Dittrich et al., 2013). This relatively small effect size and a couple of studies showing that other small adjustments of the experimental setting influence the joint Simon effect let one conclude that the joint Simon effect is very sensitive to setting and task adjustments in general (Guagnano et al., 2010; Dittrich et al., 2012, 2013; Lien et al., 2016; Stenzel and Liepelt, 2016b).

For instance, Dittrich et al. (2013) observed a Simon effect by emphasizing the spatial dimension (correspondence of response button and seat position). We followed this approach by placing the response button and the participant’s seat position on the right side of middle axis of the screen. This could also have stressed the spatial dimension in both the cat present and cat absent condition to result in a spatial coding of the participant’s actions. Lugli et al. (2015) further systematically altered the seating position in a joint Simon task with two actors after a training phase. Results showed that the seating position is even more important to the rise of a Simon effect than the spatial compatibility of stimulus and response button. Thus, the positioning of response button and participant’s seat might also contribute to the finding of an omnipresent Simon effect in the current study.

Furthermore, findings from Stenzel and Liepelt (2016b) showed that the response mode is more influential for the joint Simon effect than the attributes of the object placed next to the participant. Thus, having an object or co-actor in a turn-taking response mode results in a larger joint Simon effect than a continuously waving Japanese cat. Thus, a continuously Japanese waving cat might not be sufficient to evoke an enhanced joint Simon effect under all circumstances. In a paradigm, similar to the one used by Dolk et al. (2013a), a Japanese cat might bring about a Simon effect. In paradigms in which an individual Simon effect is already present, the Japanese cat does not exert enough influence to modulate the already existing Simon effect, neither in behavioral nor in electrophysiological measures. This is in line with findings of Lien et al. (2016) showing that the presence of a Japanese waving cat did not modulate the size of the standard Simon effect.

Nevertheless, the present study has the limitation that we did not include a human co-actor condition (e.g., Sebanz et al., 2006) to directly compare object induced and human induced Simon effects. Further, there was no control condition in which either subject and cat changed positions or in which the experimenter changed position (from left to right) to clarify whether the cat or the experimenter function as a stronger reference frame. Although combining all those conditions in a single study using a within-subject design might cause undesired effects of fatigue or lacking attention due to the required length of the experiment, further research should address these needs by suitable experimental designs, e.g., between-subject designs. An enlarged series of experiments to cover all control-conditions is also conceivable.

All in all, considering the small effect size of the Joint Simon effect and the evidence attesting its high sensitivity for experimental setup changes, it is most likely that – in our case - minimal experimental setup differences to Dolk et al. (2013a) led to an omnipresent Simon effect. Thus, we argue that the EEG experimenter caused the Simon effect independent of cat presence in our experiments. Nevertheless, based on our findings we were not able to provide evidence that a social co-actor and a salient object elicit the same ERP effect and neuronal process. However, our findings suggest that attention to other event-producing humans or objects may be an important factor for future research on joint action. Further, our findings suggest caution where to position the examiner, which might unintentionally influence experimental outcomes.

## Author Contributions

RM: data collection, data analysis, drafts and revisions of manuscript, and study design. RL: drafts and revisions of manuscript and study design. JB: drafts and revisions of manuscript, study design, and data analysis.

## Conflict of Interest Statement

The authors declare that the research was conducted in the absence of any commercial or financial relationships that could be construed as a potential conflict of interest.

## Footnotes

1 An *a priori* power analysis was conducted with G^∗^Power 3.0 using the effect sizes reported in the study of Dolk et al. (2013a), indicating 23 subjects for targeting sufficient statistical power of 0.90 at an alpha level of 0.05.

2 To control for order effects, the same ANOVA with the additional factor *order of presentation* (cat presented in first half of experiment vs. cat presented in second half of experiment) only revealed a significant interaction *cat presence* ×*order of presentation*, *F*(1,20) = 43.41, *p* < 0.001, ${\text{η}}_{\text{g}}^{2}$ = 0.07, with faster RT for the cat absent condition when it was presented in the first half (295 ms, *SD* = 10.5; vs. cat present: 311 ms, *SD* = 10.7) and faster RT in the cat present condition when it was presented in the second half (286 ms, *SD* = 13.4; vs. cat absent: 306 ms, *SD* = 13.6). The main effect *order of presentation*, the interaction *order of presentation* ×*compatibility*, both *F*(1,20) < 1, and *order of presentation* ×*cat presence* ×*compatibility*, *F*(1,20) = 2.56, *p* = 0.125, were not significant.

3 The same ANOVA including the factor *order of presentation* did not show a main effect of *order of presentation*, *F*(1,19) < 1, no interaction of *order of presentation* ×*compatibility*, *F*(1,19) < 1, no interaction of *order of presentation* ×*cat presence*, *F*(1,19) = 2.15, *p* = 0.159, and no three-way interaction of *order of presentation* ×*cat presence* ×*compatibility*, *F*(1,19) = 1.95, *p* = 0.178.

## References

[B1] Bokura H. Yamaguchi S. Kobayashi S. (2001). Electrophysiological correlates for response inhibition in a Go/NoGo task. 112 2224–2232. 10.1016/S1388-2457(01)00691-5 11738192

[B2] Bullmore E. T. Suckling J. Overmeyer S. Rabe-Hesketh S. Taylor E. Brammer M. J. (1999). Global, voxel, and cluster tests, by theory and permutation, for a difference between two groups of structural MR images of the brain. 18 32–42. 10.1109/42.750253 10193695

[B3] De Jong R. Liang C.-C. Lauber E. (1994). Conditional and unconditional automaticity: a dual-process model of effects of spatial stimulus-response correspondence. 20 731–750. 10.1037/0096-1523.20.4.731 8083631

[B4] Dittrich K. Bossert M. L. Rothe-Wulf A. Klauer K. C. (2017). The joint flanker effect and the joint Simon effect: on the comparability of processes underlying joint compatibility effects. 70 1808–1823. 10.1080/17470218.2016.1207690 27357224

[B5] Dittrich K. Dolk T. Rothe-Wulf A. Klauer K. C. Prinz W. (2013). Keys and seats: spatial response coding underlying the joint spatial compatibility effect. 75 1725–1736. 10.3758/s13414-013-0524-z 23896690

[B6] Dittrich K. Rothe A. Klauer K. C. (2012). Increased spatial salience in the social Simon task: a response-coding account of spatial compatibility effects. 74 911–929. 10.3758/s13414-012-0304-1 22528612

[B7] Dolk T. Hommel B. Colzato L. S. Schütz-Bosbach S. Prinz W. Liepelt R. (2011). How “Social” is the social Simon effect? 2:84. 10.3389/fpsyg.2011.00084 21687453 PMC3110342

[B8] Dolk T. Hommel B. Colzato L. S. Schütz-Bosbach S. Prinz W. Liepelt R. (2014). The joint Simon effect: a review and theoretical integration. 5:974. 10.3389/fpsyg.2014.00974 25249991 PMC4155780

[B9] Dolk T. Hommel B. Prinz W. Liepelt R. (2013a). The (not so) social Simon effect: a referential coding account. 39 1248–1260. 10.1037/a0031031 23339346

[B10] Dolk T. Liepelt R. Prinz W. Fiehler K. (2013b). Visual experience determines the use of external reference frames in joint action control. 8:e59008. 10.1371/journal.pone.0059008 23536848 PMC3594222

[B11] Dolk T. Prinz W. (2016). “What it takes to share a task: sharing versus shaping task representations,” in , eds Obhi S. S. Cross E. S. (Cambridge: Cambridge University Press), 3–21. 10.1017/CBO9781107279353.002

[B12] Falkenstein M. Koshlykova N. A. Kiroj V. N. Hoormann J. Hohnsbein J. (1995). Late ERP components in visual and auditory Go/Nogo tasks. 96 36–43. 10.1016/0013-4694(94)00182-K 7530187

[B13] Fallgatter A. J. Strik W. K. (1999). The NoGo-anteriorization as a neurophysiological standard-index for cognitive response control. 32 233–238. 10.1016/S0167-8760(99)00018-5 10437634

[B14] Groppe D. M. Urbach T. P. Kutas M. (2011). Mass univariate analysis of event-related brain potentials/fields I: a critical tutorial review. 48 1711–1725. 10.1111/j.1469-8986.2011.01273.x 21895683 PMC4060794

[B15] Guagnano D. Rusconi E. Umiltà C. A. (2010). Sharing a task or sharing space? On the effect of the confederate in action coding in a detection task. 114 348–355. 10.1016/j.cognition.2009.10.008 19914615

[B16] Hedge A. Marsh N. W. (1975). The effect of irrelevant spatial correspondence on two- choice response-time. 39 427–439. 10.1016/0001-6918(75)90041-4 1199779

[B17] Hommel B. (1996). S-R compatibility effects without response uncertainty. 49A, 546–571. 10.1080/027249896392496

[B18] Hommel B. Colzato L. S. van den Wildenberg W. P. M. (2009). How social are task representations? 20 794–798. 10.1111/j.1467-9280.2009.02367.x 19493327

[B19] Hommel B. Müsseler J. Aschersleben G. Prinz W. (2001). The theory of event coding (TEC): a framework for perception and action planning. 24 849–937. 10.1017/S0140525X01000103 12239891

[B20] Iani C. Anelli F. Nicoletti R. Arcuri L. Rubichi S. (2011). The role of group membership on the modulation of joint action. 211 439–445. 10.1007/s00221-011-2651-x 21472442

[B21] Ille N. Berg P. Scherg M. (2002). Artifact correction of the Ongoing EEG using spatial filters based on artifact and brain signal topographies. 19 113–124. 10.1097/00004691-200203000-00002 11997722

[B22] James W. (1890). , Vol. 1.2. New York, NY: Holt. 10.1037/10538-000

[B23] Kiernan D. Ray W. Welsh T. N. (2012). Inverting the joint Simon effect by intention. 19 914–920. 10.3758/s13423-012-0283-1 22718258

[B24] Klempova B. Liepelt R. (2017). Barriers to success: physical separation optimizes event- file retrieval in shared workspaces. 10.1007/s00426-017-0886-2 [Epub ahead of print]. 28689319

[B25] Knoblich G. Sebanz N. (2006). The Social nature of perception and action. 15 99–104. 10.1111/j.0963-7214.2006.00415.x

[B26] Kok A. (2001). On the utility of P3 amplitude as a measure of processing capacity. 38 557–577. 10.1017/S0048577201990559 11352145

[B27] Kornblum S. Hasbroucq T. Osman A. (1990). Dimensional overlap: cognitive basis for stimulus-response compatibility—A model and taxonomy. 97 253–270. 10.1037/0033-295X.97.2.253 2186425

[B28] Kutas M. McCarthy G. Donchin E. (1977). Augmenting mental chronometry: the P300 as a measure of stimulus evaluation time. 197 792–795. 10.1126/science.887923 887923

[B29] Leuthold H. (2011). The Simon effect in cognitive electrophysiology: a short review. 136 203–211. 10.1016/j.actpsy.2010.08.001 20828671

[B30] Lien M.-C. Pedersen L. Proctor R. W. (2016). Stimulus-response correspondence in go- nogo and choice tasks: are reactions altered by the presence of an irrelevant salient object? 80 912–934. 10.1007/s00426-015-0699-0 26318437

[B31] Liepelt R. (2014). Interacting hands: the role of attention for the joint Simon effect. 5:1462. 10.3389/fpsyg.2014.01462 25566140 PMC4269294

[B32] Liepelt R. Wenke D. Fischer R. (2013). Effects of feature integration in a hands-crossed version of the Social Simon paradigm. 77 240–248. 10.1007/s00426-012-0425-0 22349886

[B33] Liepelt R. Wenke D. Fischer R. Prinz W. (2011). Trial-to-trial sequential dependencies in a social and non-social Simon task. 75 366–375. 10.1007/s00426-010-0314-3 21085984

[B34] Lotze R. H. (1852). Leipzig: Weidmann’sche Buchhandlung.

[B35] Lu C.-H. Proctor R. W. (1995). The influence of irrelevant location information on performance: a review of the Simon and spatial Stroop effects. 2 174–207. 10.3758/BF03210959 24203654

[B36] Lugli L. Iani C. Milanese N. Sebanz N. Rubichi S. (2015). Spatial parameters at the basis of social transfer of learning. 41 840–849. 10.1037/xhp0000047 25867503

[B37] Magliero A. Bashore T. R. Coles M. G. H. Donchin E. (1984). On the dependence of P300 latency on stimulus evaluation processes. 21 171–186. 10.1111/j.1469-8986.1984.tb00201.x 6728983

[B38] Miller J. E. Carlson L. A. Hill P. L. (2011). Selecting a reference object. 37 840–850. 10.1037/a0022791 21417510

[B39] Morrey R. D. (2008). Confidence intervals from normalized data: a correction to Cousineau (2005). 4 61–64. 10.20982/tqmp.04.2.p061

[B40] Müller B. C. N. Brass M. Kühn S. Tsai C.-C. Nieuwboer W. Dijkerhuis A. (2011). When Pinocchio acts like a human, a wooden hand becomes embodied. Action co-representation for non-biological agents. 49 1373–1377. 10.1016/j.neuropsychologia.2011.01.022 21241722

[B41] Nicoletti R. Umiltà C. (1994). Attention shifts produce spatial stimulus codes. 56 144–150. 10.1007/BF00419701 8008776

[B42] Porcu E. Bölling L. Lappe M. Liepelt R. (2016). Pointing out mechanisms underlying joint action. 78 972–977. 10.3758/s13414-016-1093-8 27016344

[B43] Prinz W. (1997). Perception and action planning. 9 129–154. 10.1080/713752551

[B44] Prinz W. (2005). “An ideomotor approach to imitation,” in , ed. Hurley S. (Cambridge, MA: MIT Press), 141–156.

[B45] Puffe L. Dittrich K. Klauer K. C. (2017). The influence of the Japanese waving cat on the joint spatial compatibility effect: a replication and extension of Dolk, Hommel, Prinz, and Liepelt (2013). 12:e0184844. 10.1371/journal.pone.0184844 28910413 PMC5599021

[B46] Ragot R. Renault B. (1981). P300 as a function of S-R compatibility and motor programming. 13 289–294. 10.1016/0301-0511(81)90044-2 7342999

[B47] Renault B. Fiori N. Giami S. (1988). Latencies of event related potentials as a tool for studying motor processing organization. 26 217–230. 10.1016/0301-0511(88)90021-X 3207784

[B48] Roberts L. E. Rau H. Lutzenberger W. Birbaumer N. (1994). Mapping P300 waves onto inhibition: go/Nogo discrimination. 92 44–45. 10.1016/0168-5597(94)90006-x 7508852

[B49] Ruys K. I. Aarts H. (2010). When competition merges people’s behavior: interdependency activates shared action representations. 46 1130–1133. 10.1016/j.jesp.2010.05.016

[B50] Sebanz N. Knoblich G. Prinz W. (2003). Representing others’ actions: just like one’s own? 88 B11–B21. 10.1016/S0010-0277(03)00043-X 12804818

[B51] Sebanz N. Knoblich G. Prinz W. Wascher E. (2006). Twin peaks: an ERP study of action planning and control in coacting individuals. 18 859–870. 10.1162/jocn.2006.18.5.859 16768383

[B52] Sebanz N. Knoblich G. Stumpf L. Prinz W. (2005). Far from action-blind: representation of others’ actions in individuals with autism. 22 433–454. 10.1080/02643290442000121 21038260

[B53] Sellaro R. Dolk T. Colzato L. S. Liepelt R. Hommel B. (2015). Referential coding does not rely on location features: evidence for a nonspatial joint Simon effect. 41 186–195. 10.1037/a0038548 25528013

[B54] Sellaro R. Treccani B. Rubichi S. Cubelli R. (2013). When co-action eliminates the Simon effect: disentangling the impact of co-actor’s presence and task sharing on joint-task performance. 4:844. 10.3389/fpsyg.2013.00844 24312066 PMC3833097

[B55] Simon J. R. Rudell A. P. (1967). Auditory S-R compatibility: the effect of an irrelevant cue on information processing. 51 300–304. 10.1037/h0020586 6045637

[B56] Stenzel A. Chinellato E. Bou M. A. T. del Pobil ÁP. Lappe M. Liepelt R. (2012). When humanoid robots become human-like interaction partners: corepresentation of robotic actions. 38 1073–1077. 10.1037/a0029493 22866762

[B57] Stenzel A. Chinellato E. del Pobil ÁP. Lappe M. Liepelt R. (2013). How deeply do we include robotic agents in the self. 10 1–13. 10.1142/S0219843613500151

[B58] Stenzel A. Liepelt R. (2016a). Joint action changes valence-based action coding in an implicit attitude task. 80 889–903. 10.1007/s00426-015-0684-7 26215432

[B59] Stenzel A. Liepelt R. (2016b). Joint Simon effects for non-human co-actors. 78 143–158. 10.3758/s13414-015-0994-2 26486640

[B60] Tekok-Kilic A. Shucard J. L. Shucard D. W. (2001). Stimulus modality and go/nogo effects on P3 during parallel visual and auditory continuous performance tasks. 38 578–589. 10.1017/S0048577201991279 11352146

[B61] Tsai C.-C. Brass M. (2007). Does the human motor system simulate Pinocchio’s actions? Coacting with a human hand versus a wooden hand in a dyadic interaction. 18 1058–1062. 10.1111/j.1467-9280.2007.02025.x 18031412

[B62] Tsai C.-C. Kuo W.-J. Hung D. L. Tzeng O. J. L. (2008). Action co-representation is tuned to other humans. 20 2015–2024. 10.1162/jocn.2008.20144 18416679

[B63] Tsai C.-C. Kuo W.-J. Jing J.-T. Hung D.-L. Tzeng O. J.-L. (2006). A common coding framework in self–other interaction: evidence from joint action task. 175 353–362. 10.1007/s00221-006-0557-9 16799815

[B64] Valle-Inclán F. (1996). The locus of interference in the Simon effect: an ERP study. 43 147–162. 10.1016/0301-0511(95)05181-3 8805969

[B65] van der Wel R. P. R. D. Sebanz N. Knoblich G. (2016). “A joint action perspective on embodiment,” in , eds Coello Y. Fischer M. (Oxford: Psychology Press), 165–181.

[B66] Verleger R. Jaskowski P. Wascher E. (2005). Evidence for an integrative role of P3b in linking reaction to perception. 19 165–181. 10.1027/0269-8803.19.3.165

[B67] Vlianic E. Liepelt R. Colzato L. S. Prinz W. Hommel B. (2010). The virtual co-actor: the social Simon effect does not rely on online feedback from the other. 1:208. 10.3389/fpsyg.2010.00208 21833264 PMC3153814

[B68] Wascher E. Wauschkuhn B. (1996). The interaction of stimulus- and response-related processes measured by event-related lateralizations of the EEG. 99 149–162. 10.1016/0013-4694(96)95602-3 8761051

[B69] Weeks D. J. Proctor R. W. Beyak B. (1995). Stimulus–Response compatibility for vertically oriented stimuli and horizontally oriented responses: evidence for spatial coding. 48 367–383. 10.1080/14640749508401395 7610272

[B70] Welsh T. N. Kiernan D. Neyedli H. F. Ray M. Pratt J. Potruff A. (2013a). Joint Simon effects in extrapersonal space. 45 1–5. 10.1080/00222895.2012.746635 23387518

[B71] Welsh T. N. Kiernan D. Neyedli H. F. Ray M. Pratt J. Weeks D. J. (2013b). On mechanisms, methods, and measures: a response to Guagnano, Rusconi, and Umiltà. 45 9–14. 10.1080/00222895.2012.746560

[B72] Zhou B. Zhang J. X. Tan L. H. Han S. (2004). Spatial congruence in working memory: an ERP study. 15 2795–2799. 15597057

